# Reactive oxygen species dependent phosphorylation of the liver kinase B1/AMP activated protein kinase/ acetyl-CoA carboxylase signaling is critically involved in apoptotic effect of lambertianic acid in hepatocellular carcinoma cells

**DOI:** 10.18632/oncotarget.19592

**Published:** 2017-07-26

**Authors:** Arong Jeong, Ju-Ha Kim, Hyo-Jung Lee, Sung-Hoon Kim

**Affiliations:** ^1^ Cancer Molecular Target Herbal Research Center, College of Korean Medicine, Kyung Hee University, Seoul 131-701, Republic of Korea

**Keywords:** lambertianic acid, hepatocellular carcinoma, apoptosis, AMPK, ROS

## Abstract

Though lambertianic acid (LA) is reported to have hypolipidemic activity in liver, its underlying anticancer mechanism is poorly understood so far. Thus, in the present study, apoptotic mechanism of LA was elucidated in HepG2 and SK-Hep1 hepatocellular carcinoma (HCC) cells. Here LA increased cytotoxicity, sub-G1 population and Annexin V/PI positive cells in two HCC cells. Also, LA cleaved caspase-3 and poly(ADP-ribose) polymerase (PARP), activated phosphorylation of liver kinase B1 (LKB1)/AMP activated protein kinase (AMPK)/ acetyl-CoA carboxylase (ACC) pathway and also suppressed antiapoptotic proteins such as phosphorylation of Akt/ mammalian target of rapamycin (mTOR) and the expression of B cell lymphoma-2 (Bcl-2)/ B-cell lymphoma-extra large (Bcl-xL) and cyclooxygenase-2 (COX-2) in two HCC cells. Furthermore, LA generated reactive oxygen species (ROS) in HepG2 cells and AMPK inhibitor compound C or ROS inhibitor N-acetyl-L-cysteine (NAC) blocked the apoptotic ability of LA to cleave PARP or increase sub G1 population in HepG2 cells. Consistently, cleavages of PARP and caspase-3 were induced by LA only in *AMPK*^*+/+*^ MEF cells, but not in *AMPK*^*-/-*^ MEF cells. Also, immunoprecipitation (IP) revealed that phosphorylation of LKB1/AMPK through their binding was enhanced in LA treated HepG2 cells. Overall, these findings suggest that ROS dependent phosphorylation of LKB1/AMPK/ACC signaling is critically involved in LA induced apoptosis in HCCs.

## INTRODUCTION

Hepatocellular carcinoma (HCC) is approximately 90% of all cases of primary liver cancer, the 5th most common cancer worldwide and the third leading cause of cancer-related mortality [1, 2]. The main risk factors include hepatitis B and C virus infection, alcohol–related liver cirrhosis, non-alcoholic steatohepatitis and ingestion of aflatoxin B1 [3]. Patients with early-stage of HCC are amenable to therapies such as resection, liver transplantation or local ablation [4]. Nevertheless, less than 20% of patients are eligible for curative treatment including chemoembolization or sorafenib because of its late-stage, multiple comorbidities and hepatic dysfunction [5]. Also, even sorafenib has not been widely used because of its high cost and toxicity [6]. Thus, recently natural compunds are attractive for combination therapy or novel target therapy for HCC.

AMP-activated protein kinase (AMPK) is a key regulator of energy metabolism [7] by activating the synthesis of fatty acid, cholesterol, protein through phosphorylation of metabolic enzymes and ATP-generating processes, including glucose uptake [8]. A tumor suppressor liver kinase B1 (LKB1) is its upstream kinase that phosphorylates and activates AMPK signaling by encoding serine/threonine kinase [9, 10]. Also, many studies revealed that AMPK suppresses cell proliferation by the inhibition of cell cycle progression and regulation of mitosis [11, 12]. Thus, AMPK activation is a therapeutic target for the prevention and treatment of cancer by metformin and other anticancer agents.

Though many researchers have been trying to find magic bullets for cancer therapy for decades, recent attention moves toward to cancer prevention rather than treatment with no magic bullets for cancer fighting to date. Thus, it was well documented that several compounds from natural products have cancer preventive efficacies, such as curcumin [13–16], capsaicin [17, 18], resveratrol [19–22], ursolic acid [23], brazilin [24], isothiocyanates [25], tanshinone I [26], tanshinone IIa [27], coumestrol [28], ginkgetin [29], emodin [30] and auraptene [31–39].

In the same line, though labmertianic acid (LA) is known to have anti-obesity [40], stress-protective [41], anti-allergic [42] and neurotropic [43, 44] activities, its other anti-cancer studies have not been reported except our group’s report on anticancer effect of LA via androgen receptor(AR) ablation [45] inhibition until now. Thus, in the current study, the underlying apoptotic mechanism of LA was elucidated in association with reactive oxygen species(ROS) and liver kinase B1 (LKB1)/AMP activated protein kinase (AMPK)/acetyl-CoA carboxylase (ACC) signaling pathway in HCCs.

## RESULTS

### Cytotoxic effect of LA in human hepatocellular carcinoma (HCC) cells

**Figure 1:**
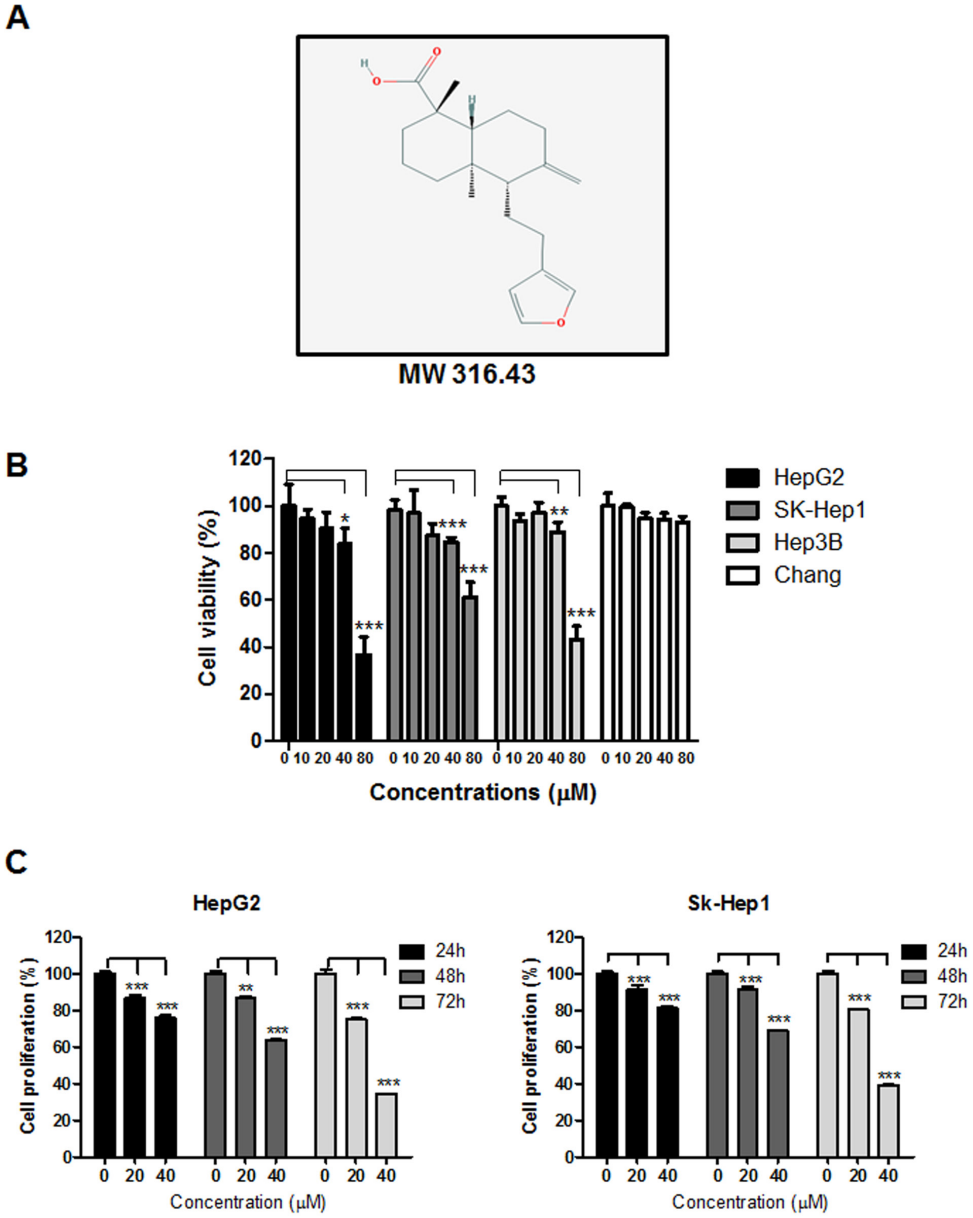
Effect of LA on cytotoxicity in HepG2, SK-Hep1, Hep3B and Chang cells **(A)** Chemical structure of LA, **(B)** cytotoxic effect of LA in HCCs and Chang liver normal cells. Cells were seeded onto 96 well microplates and treated with various concentrations of LA (0, 10, 20, 40, 80 μM) for 24 h. Cell viability was evaluated by MTT assay. **(C)** Effect of LA on proliferation of HepG2 and SK-Hep1 cells. Cells were cultured with LA(0-40 μM) for 24h, 48h or 72 h and then stained with crystal violet. Data represent means ± SD. *, p<0.05, **, p<0.01 and ***, p<0.001 versus untreated control (n=3, one-way ANOVA, Tukey's test).

The cytotoxicity of LA in HepG2 and SK-Hep1 and Hep3B hepatocellular carcinoma (HCC) cells and Chang hepatocytes was evaluated by using MTT assay. Cells were treated with indicated concentrations of LA (0, 10, 20, 40, 80 μM) for 24 h. As shown in Figure 1, LA significantly suppressed the viability of HepG2, SK-Hep1 and Hep3B cells in a concentration dependent fashion, but not Chang normal hepatocyte cells. However, we used noninvasive and lipidemic HepG2 cells and metastatic SK-Hep1 cells origianted from hepatoblastoma of white male rather than invasive Hep 3B cells originated from HCC of black male in next experiments [46], though the susceptibility of Hep 3 B cells to LA was almost similar to that of SK-Hep1 cells. Next, cell proliferation assay was conducted in HepG2 and SK-Hep1 cells using crystal violet staining. After exposure to LA for 24 h, 48 h and 72 h, LA significantly inhibited proliferation of two HCC cells in a concentration and time dependent manner (Figure 1C).

### LA increased sub G1 population and regulated apoptosis related proteins in HCC cells

**Figure 2:**
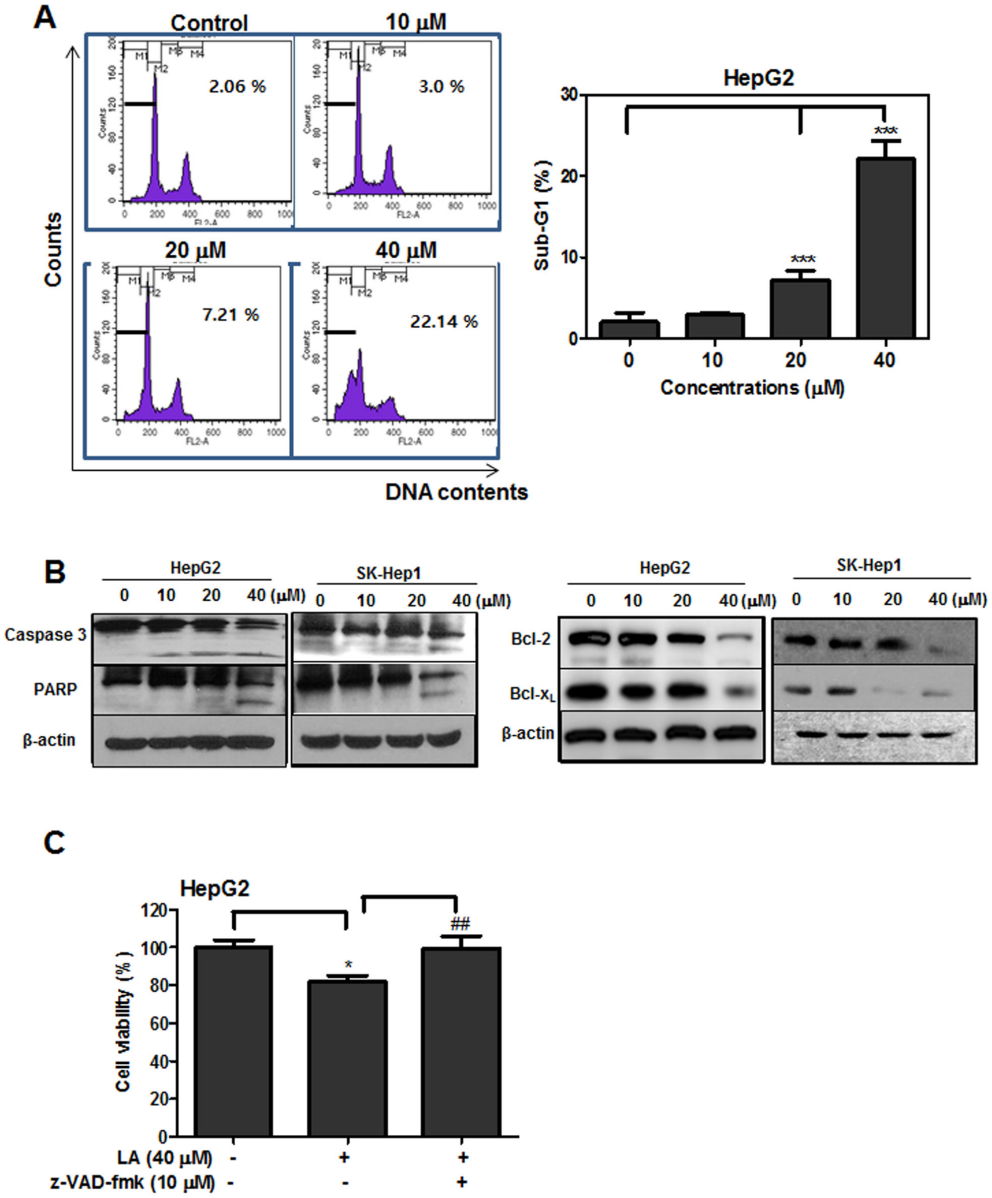
Effect of LA on sub-G1 population and apoptosis related proteins in hepatocellular carcinoma cells **(A)** Effect of LA on sub-G1 population in HepG2 cells. HepG2 cells were exposed to LA (10, 20, 40 μM) for 24 h. After fixing in 75% ethanol, the cells were stained with propidium iodide and the cell cycle was analyzed by flow cytometry. Bar graphs showed quantification of sub-G1 cell population (%). Data represent means ± SD. **, p<0.01 versus untreated control. **(B)** Effect of LA on sub-G1 population and apoptosis related proteins in HCCs.HepG2 and SK-Hep1 cells were treated with various concentrations of LA for 24 h and subjected to Western blotting for caspase-3, PARP, Bcl-2, Bcl-xL and β-actin. **(C)** Effect of pancaspase inhbibtor Z-VAD-fmk on the viability of LA treated HepG2 cells. HepG2 cells were treated with 40 μM of LA with or without Z-VAD-fmk (10 μM). The cell viability was measured by MTT. Data represent means ± SD. *, p<0.05 and ***, p<0.001 versus untreated control. ##, p<0.01 versus LA (40 μM) treated group (n=3, one-way ANOVA, Tukey's test).

To test whether the cytotoxic effect of LA is due to apoptosis induction, cell cycle analysis and Western blotting were performed. LA increased sub-G1 in a dose-dependent fashion. Especially, LA at 40 μM elevated the sub-G1 population to 22.14% compared to untreated control (2.06%) (Figure 2A). Also, Western blotting showed that LA cleaved caspase-3, PARP and suppressed Bcl-2, Bcl-xL (Figure 2B) in HepG2 and SK-Hep1 cells. However, pan-caspase inhibitor Z-VAD-fmk reversed the cytotoxicity induced by LA in HepG2 cells (Figure 2C).

### LA activated phosphorylation of LKB1/AMPK/ACC signaling in HCC cells

**Figure 3:**
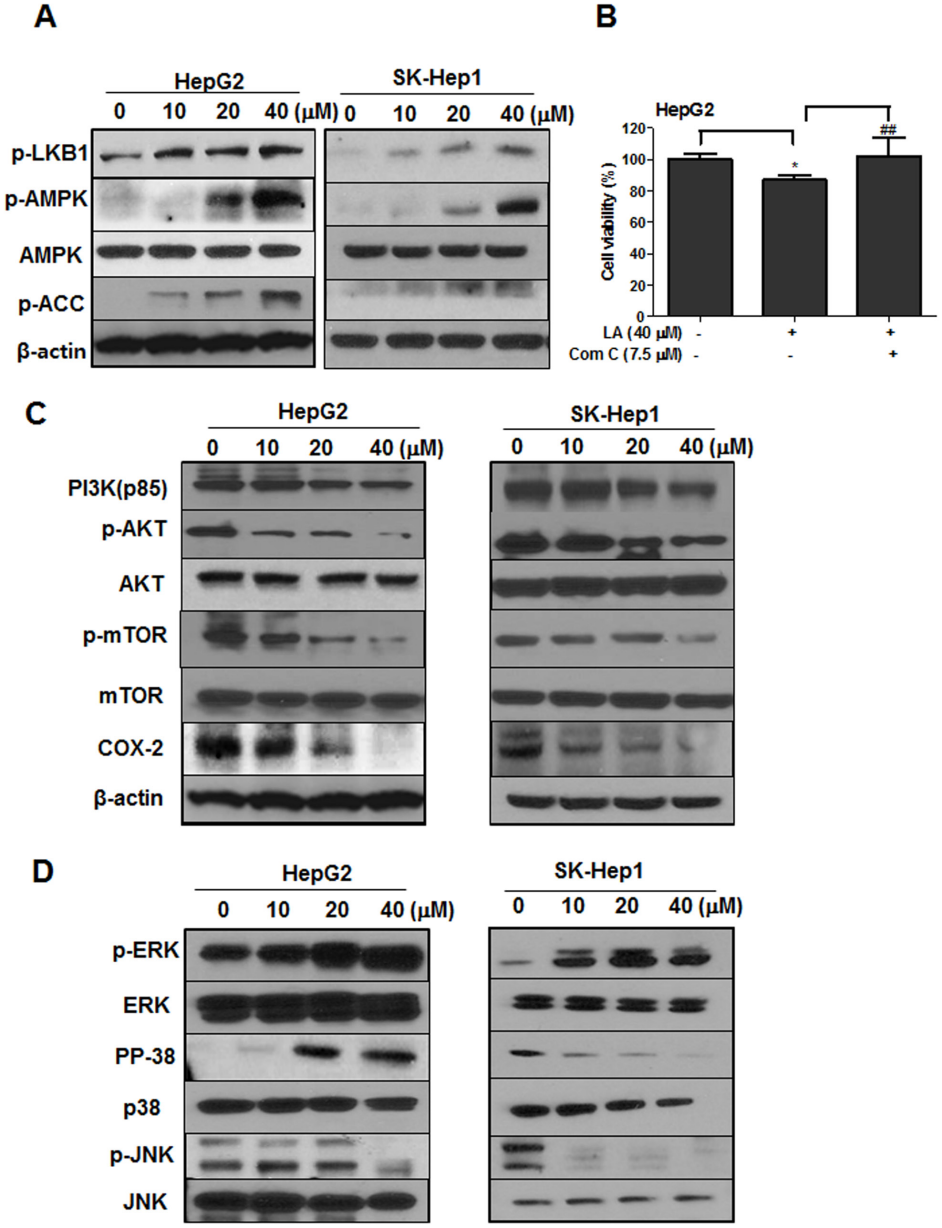
Effect of LA on AMPK and its related proteins in HCC cells **(A)** HepG2 and SK-Hep1 cells were treated with indicated concentrations of LA for 24 h and the cells were subjected to Western blotting for p-LKB1, p-AMPK, AMPK, p-ACC, COX-2 and β-actin. **(B)** HepG2 cells were treated with 40 μM of LA with or without 7.5 μM of compound C for 24 h and MTT assay was conducted. Data represent means ± SD. *, p<0.05 versus untreated control. ##, p<0.01 versus LA (40 μM) only treated group (n=3, one-way ANOVA, Tukey's test). **(C)** HepG2 and SK-Hep1 cells were treated with various concentrations of LA and Western blotting was conducted with antibodies of PI3K, p-AKT, AKT, p-mTOR, mTOR, COX-2 and β-actin. **(D)** HepG2 and SK-Hep1 cells were treated with various concentrations of LA and Western blotting was performed with antibodies of p-ERK, ERK, p-p38, p38, p-JNK and JNK.

To confirm whether the apoptotic effect of LA is related to AMPK signaling, Western blotting was performed in LA treated HCC cells with antibodies of p-LKB1, p-AMPK and p-ACC. Here, phosphorylation of LKB1, AMPK and ACC was increased in a dose dependent manner in LA treated HepG2 and SK-Hep1 cells (Figure 3A). As shown in Figure 3B, the cytotoxic effect of LA was significantly reversed by AMPK inhibitor compound C treatment. In addition, Western blotting showed that LA suppressed the expression of PI3K, p-AKT, p-mTOR and COX-2 in HepG2 and SK-Hep1 cells (Figure 3C). Also, as shown in Figure 3D, LA activated phosphorylation of ERK and p38 and attenuated phosphorylation of JNK in HepG2 cells. In contrast, LA decreased phosphorylation of p38 and JNK and increased phosphorylation of ERK in SK-Hep1 cells.

### LA generated ROS production in HepG2 cells

**Figure 4:**
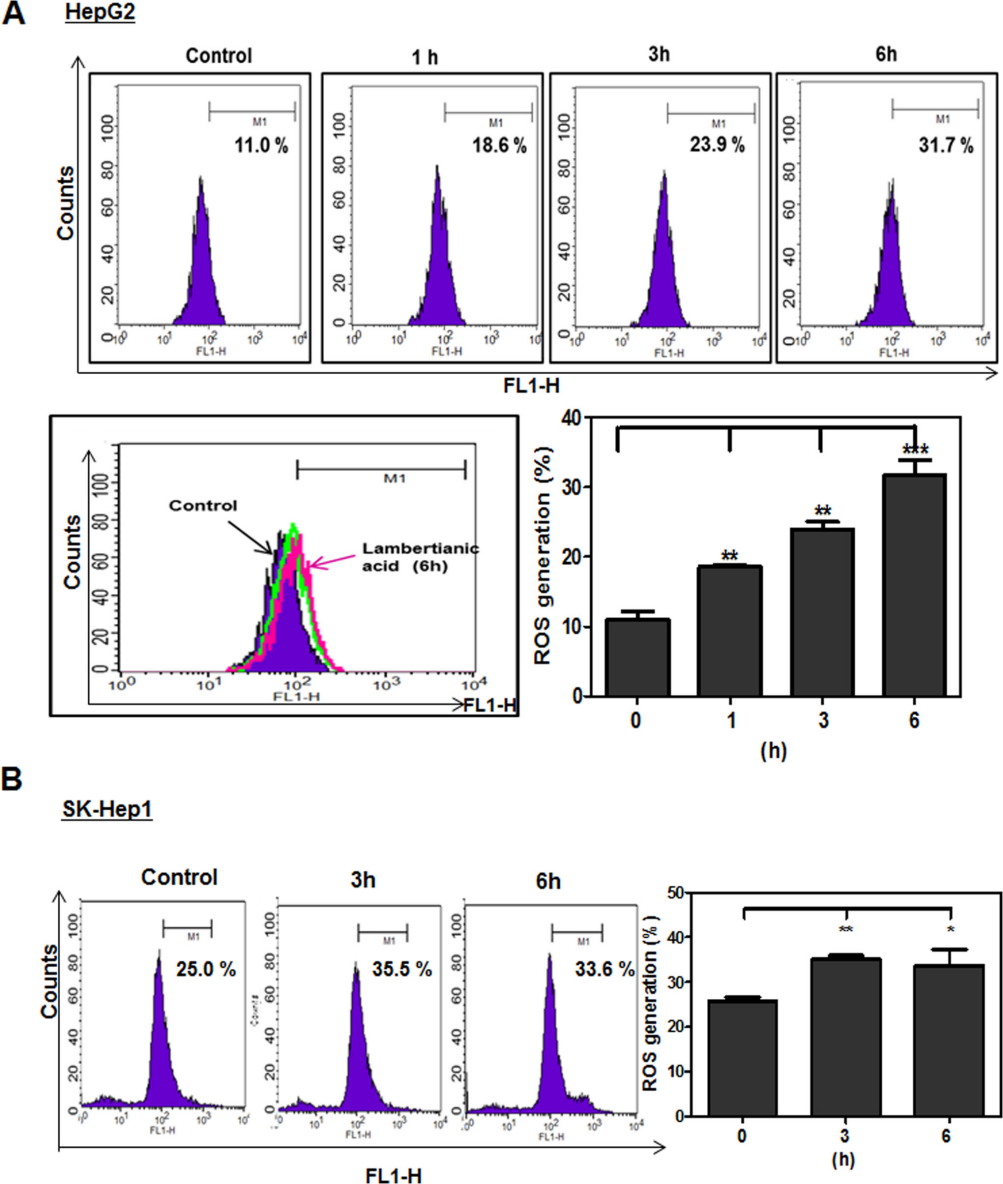
Effect of LA on ROS production in HepG2 and SK-Hep1 cells HepG2 **(A)** and SK-Hep1 **(B)** cells were treated with LA (40 μM) for 1 h, 3 h, 6 h and then 5 μM DCFH-DA for 30 min at 37°C. Experiments were performed three times. Fluorescence intensity was measured by FACS Calibur. Bar graphs showed quantification of ROS generation (%). Data represent means ± SD. * p<0.05, **, p<0.01 and ***, p<0.001 versus untreated control (n=3, one-way ANOVA, Tukey's test).

To test whether ROS play a crictial role in LA induced apoptosis, DCFH-DA staining was used to measure the ROS generation by FACS Calibur. As shown in Figure 4, LA induced ROS production in HepG2 and SK-Hep1 cells in a time dependent manner compared to untreated control.

### ROS dependent AMPK activation is critically involved in LA induced apoptosis in HepG2 and SK-Hep1 cells

**Figure 5:**
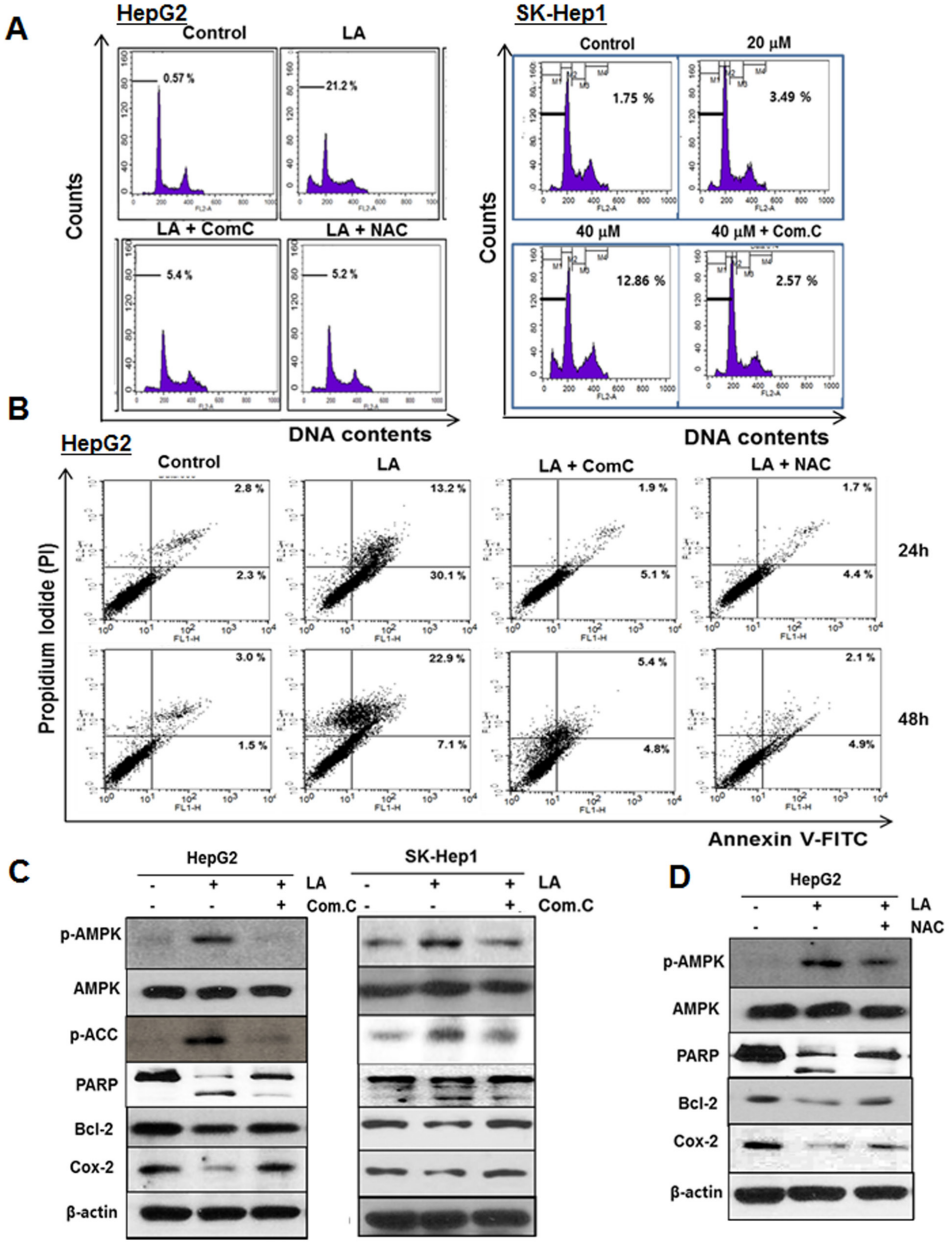
ROS and AMPK signalings mediate LA induced apoptosis in HCC cells **(A)** HepG2 and SK-Hep1 cells were treated with LA (40 μM) in the absence and presence of compound C (7.5 μM) or NAC (5 mM). After fixing in 75% ethanol, the cells were stained with PI and the cell cycle was analyzed by flow cytometry. **(B)** HepG2 cells were treated with LA (40 μM) for 24 h and 48 h in the absence and presence of compound C (7.5 μM) or NAC (5 mM). The cells were stained using FITC-Annexin V/PI dye and early and late apoptotic portions were detected by flow cytometry. **(C)** HepG2 and SK-Hep1 cells were treated with various concentrations of LA (20, 40 μM) for 24 h with or without compound C. Then Western blotting was conducted in LA and/or compound C treated HCCs with antibodies of p-AMPK, AMPK, p-ACC, PARP, Bcl-2, COX-2 and β-actin. **(D)** HepG2 cells were exposed to LA (40 μM) in the presence or absence of NAC and subjected to Western blotting for p-AMPK, AMPK, PARP, Bcl-2, COX-2 and β-actin.

Given that ROS generation is related to AMPK mediated apoptosis [47, 48], cell cycle anaylsis and Western blotting were conducted with AMPK inhibitor compound C or ROS inhibitor NAC in LA treated HepG2 cells. The increased sub-G1 population by LA was significantly attenuated in HepG2 and SK-Hep1 cells by compound C and/or NAC (Figure 5A). Consistently, Cell apoptosis assay using Annexin-V/PI double staining revealed that LA increased the percentage of early apoptotic cells (Annexin V+/PI− staining: 30.1 %) for 24 h and late apoptotic or necrosis cells (Annexin V+/PI+ staining: 22.9%) for 48h. Conversely, increased early/late apoptosis by LA was significantly reversed by compound C or NAC in HepG2 cells (Figure 5B). Likewise, the increased phosphorylation of AMPK and ACC, PARP cleavage and decreased expression of Bcl-2 and Cox-2 by LA were reversed in HepG2 and SK-Hep1 cells by compound C (Figure 5C) or in HepG2 cells by NAC (Figure 5D).

**Figure 6:**
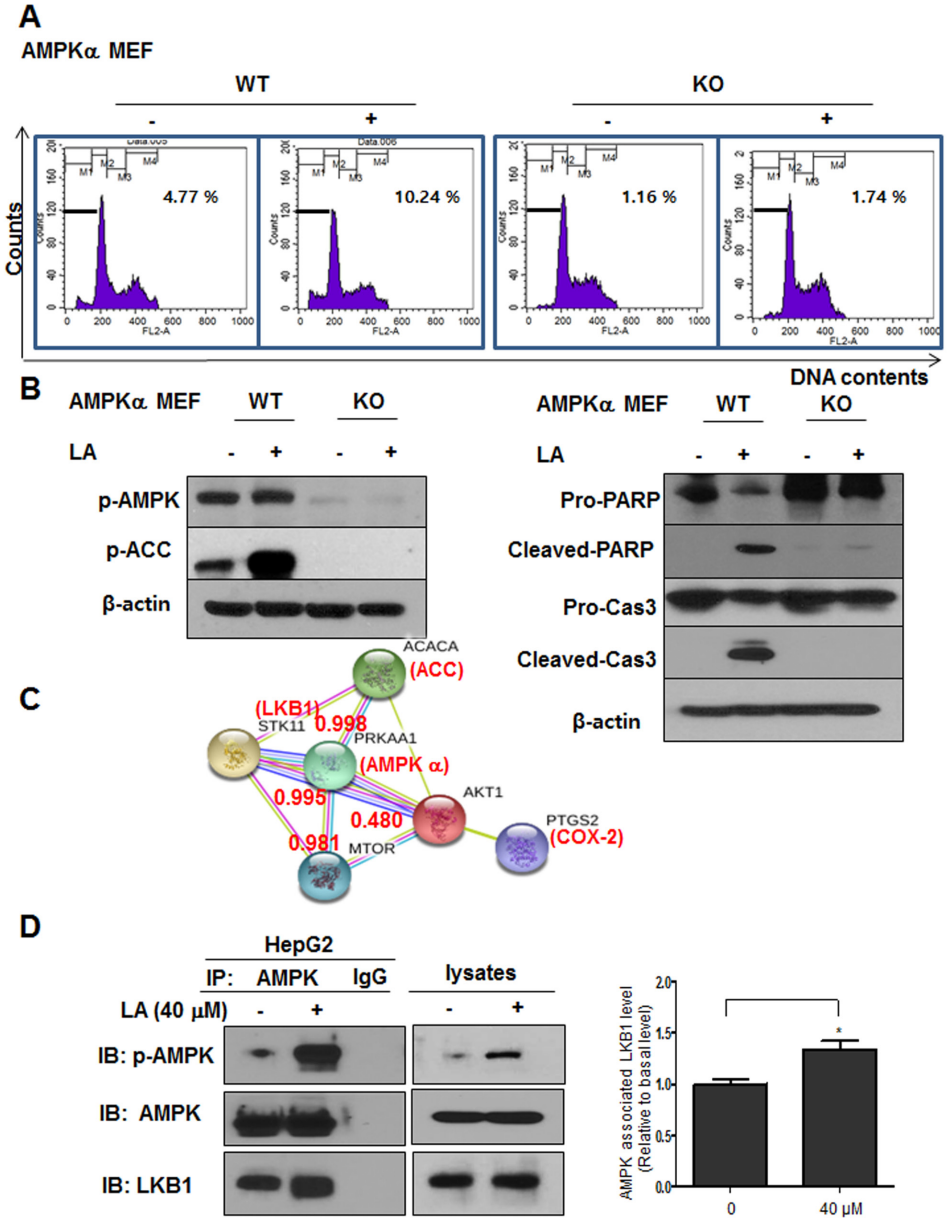
The critical role of AMPK in LA induced apoptosis in MEF cells and its interaction proteins **(A)**AMPKα wild type and AMPK knockout MEF cells were treated with LA (40 μM) for 24 h. After fixing in 75% ethanol, the cells were stained with PI and the cell cycle was analyzed by flow cytometry. **(B)** AMPKα wild type and AMPK knockout MEF cells were treated with LA (40 μM) for 24 h and subjected to Western blotting with antibodies of p-AMPK, p-ACC, PARP, Cleaved-PARP, Caspase3, Cleaved-caspase3 and β-actin. **(C)** Interacting proteins with AMPK through PPI networks by STRING database. Red text (interaction score). Different color lines represent the interaction score between AMPK and its related proteins. **(D)** Effect of LA the binding between LKB1 and AMPK in HepG2 cells. Immunoprecipitation (IP) was performed with lysates from HepG2 cells using anti-AMPK antibody and Western blotting was performed to detect AMPK and LKB1 in whole cell lysates. Bar graphs show associated level between AMPK and LKB1 in LA treated HepG2 cells. *p<0.05 versus untreated control (Student’s *t*-test).

To further confirm the critical role of AMPK in LA induced apoptosis, AMPK α wild type and knockout (KO) mouse embryonic fibroblast (MEF) cells were used in this study. Herein LA increased sub-G1 population to 10.24% in AMPK α wild type MEF cells compard to untreated control (4.77%), while there were no significant changes of subG1 population in AMPK KO MEF cells (Figure 6A). Consitently, Western blotting showed that cleavages of PARP and caspase-3 and phosphorylation of AMPK and ACC were induced in AMPK α wild type MEF cells by LA, but not in AMPK KO MEF cells (Figure 6B). Of note, STRING database showed that Protein-Protein Interaction(PPI) scores between AMPK and other proteins such as ACC, LKB1, mTOR and AKT1 were found 0.998, 0.995, 0.981 and 0.480, respectively (Figure 6C). Next, immunoprecipitation (IP) was performed with lysates from HepG2 cells using anti-AMPK antibody and Western-blot analysis was also conducted to confirm the binding of AMPK and LKB1. Here IP revealed that AMPK was directly bound to LKB1 in LA treated HepG2 cells (Figure 6D).

## DISCUSSION

Hepatocellular carcinoma (HCC) as one of the most malignant human cancers [49] is known to be caused by risk factors including hepatitis B and C virus infection, alcohol abuse, non-alcoholic fatty liver disease, aflatoxins, diabetes, obesity, and genetic factors [50]. Nevertheless, the evident therapeutics for HCC still remain unclear so far [51, 52]. Hence, the development of novel therapies for HCC has been requested all over the world.

Apoptosis is well defined as a programmed cell death; there are several types of cell death including apoptosis, pyroptosis, necrosis, or autophagy [53–55]. It is characterized by cell shrinkage, nuclear condensation and fragmentation, loss of adhesion to extracellular matrix and membrane blebbing in the cells [56]. Nowadays, apoptosis induction is recognized as one of the important strategies for cancer prevention or treatment [57, 58].

In the current study, the apoptotic mechanism of LA was elucidated in HCCs. LA suppressed the viability of three HCC cells in a dose-dependent manner. To confirm the cytotoxicity of LA was due to apoptosis induction, cell cycle analysis was conducted. LA significantly increased sub-G1 population and the number of Annexin V/PI stained cells in HepG2 and SK-Hep1 cells, indicating that cytotoxicity of LA is induced by apoptotic effect of LA [59].

The cysteine-dependent aspartate-specific proteases as caspases have pivotal roles in apoptosis induction. Caspases consist of two groups; initiator caspases (caspase-2, -8, -9 and -10) and effector caspases (caspase-3 and -7) [60]. Once the process of intrinsic apoptosis pathway is initiated, cytochrome c is released from the mitochondria, which is a peripheral protein of the mitochondrial inner membrane [61]. The released cytochrome c attaches to apoptosis-activating factor-1 (Apaf-1) and procaspase-9 to form apoptosome in cytosol [62]. After procaspase-9 is activated by apoptosome, which in turn activates procaspase-3 to caspase-3 leading to apoptosis [60]. Here, LA cleaved PARP, caspase-3 and inhibited antiapoptotic proteins such as Bcl-2, Bcl-xL and also suppressed the expression of COX-2, PI3K, p-AKT and p-mTOR as survival pathway proteins in two HCC cells, implying LA induces apoptosis via inhibition of antiapoptotic and survival proteins. Also, the apoptotic effect of LA was blocked by pan-caspase inhibitor, Z-VAD-fmk, indicating that apoptosis induced by LA is mainly via caspase activation. Also, It was well documented that MAPKs including ERK, p38 and JNK regulate multicellular functions such as proliferation, differentiation, mitosis, gene expression, cell survival and apoptosis [63]. As shown in Figure 3D, LA activated phosphorylation of ERK and p38 and attenuated phosphorylation of JNK in HepG2 cells, while it decreased phosphorylation of p38 and JNK and increased phosphorylation of ERK in SK-Hep1 cells, implying cell specific effect of LA on MAPKs and further mechanistic study in the near future.

AMPK is a serine/threonine protein kinase, a key regulator of cellular metabolism and plays an important role in apoptosis [64, 65]. Several reports have revealed that AMPK can be a potential therapeutic target for cancer treatment [66, 67]. Here, LA induced phosphorylation of AMPK, its upstream LKB1 and downstream ACC and also decreased protein expression of COX-2 in HCC cells, indicating the important role of LKB1/AMPK/ACC signaling in LA induced anticancer effect, since AMPK related signaling is critically involved in survival and proliferation of cancer cells through Warburg effect during lipid metabolism including lipogenesis [68, 69].

**Figure 7:**
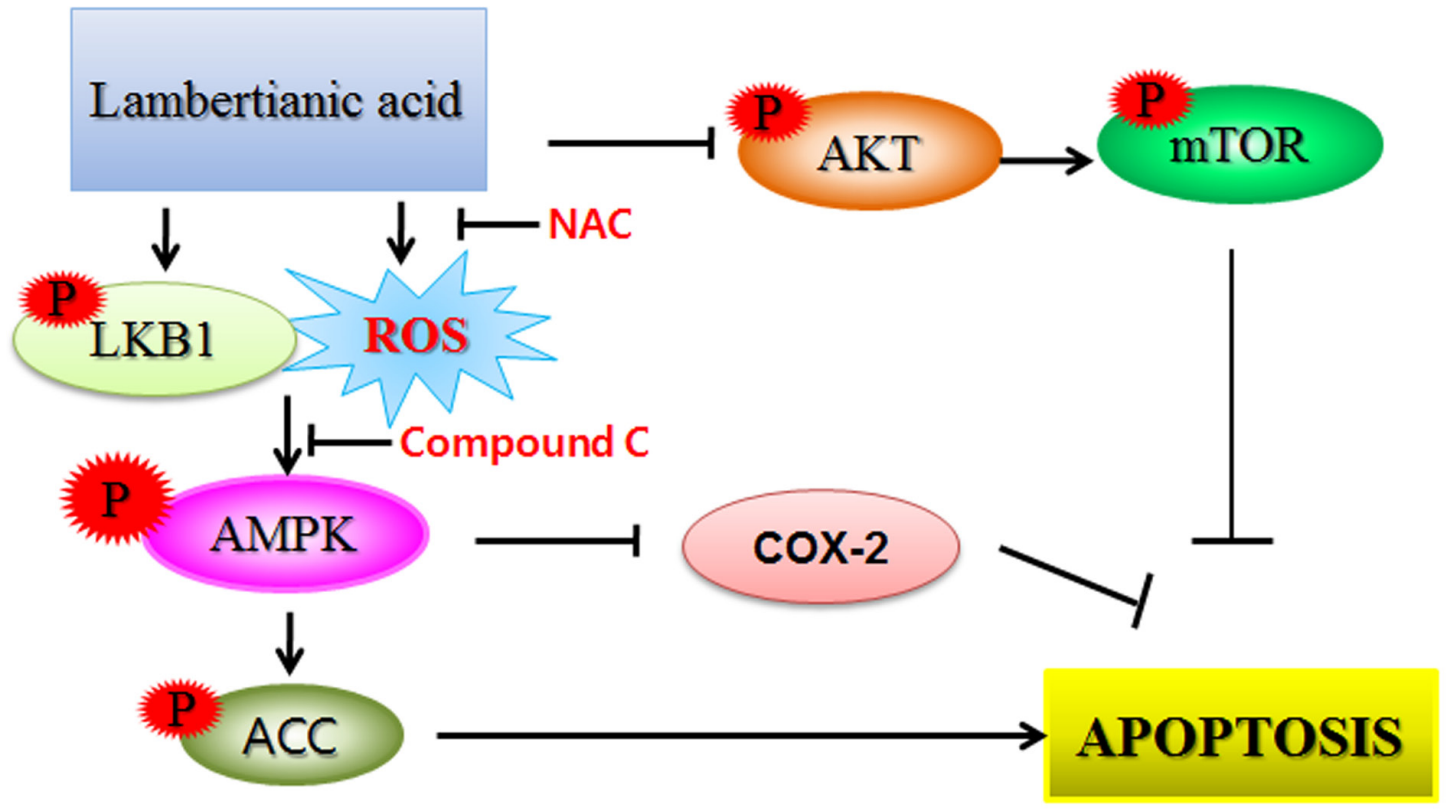
The schematic diagram for LA induced apoptosis via phosphorylation of LKB1/AMPK/ACC, ROS production and inhibition of p-AKT/mTOR signaling

It is well documented that the increased level of ROS is often observed during apoptosis induction in several cells [70]. ROS are ubiquitous among biological activities and excessive generation of ROS has been shown to induce damage in a variety of cancer cells via disruption of lipid membranes, proteins and DNA [71]. The current study revealed that LA increased ROS production in a time dependent manner. Conversely, the increase of sub G1 population and Annexin V/PI stained cells by LA was reversed by AMPK inhibitor compound C or ROS scavenger NAC in two HCC cells. Consistently, compound C blocked the ability of LA to induce phosphorylation of AMPK/ACC, PARP cleavage and decreased expression of Bcl-2 and COX-2 in two HCC cells. Also, NAC reversed phosphorylation of AMPK, PARP cleavage and decreased expression of Bcl-2 and COX-2 in HepG2 cells, demonstrating that LA generates ROS and subsequently induces AMPK phosphorylation, PARP cleavage and inhibits antiapoptotic proteins such as Bcl-2 and COX-2 leading to apoptosis in HCC cells (Figure 7).

Notably, LA increased sub-G1 population only in AMPK α wild type MEF cells, but not in AMPK KO MEF cells. Similarly, LA induced cleavages of PARP and caspase-3 and phosphorylation of AMPK/ACC in AMPK α wild type MEF cells, but not in AMPK KO MEF cells, indicating the pivotal role of LKB1/AMPK/ACC signaling in LA induced apoptosis. Overall, our findings support scientific evidence that LA can be a potent chemopreventive candidate for HCC treatment via ROS dependent phosphorylation of LKB1/AMPK/ACC signaling.

## MATERIALS AND METHODS

### Isolation of LA

Pinus koraiensis leaves (3 kg) were pulverized, immersed in 50% MeOH (10 L) for 3 days and distilled to be concentrated for 10 h by using Rotary Evaporator(IKA Korea Limited, Seoul, Korea). Then the MeOH extracts were partitioned with EtOAc / distilled water (1:1) and the water layer was suspended and partitioned with n-butanol/distilled water. A part of EtOAc fraction was subjected to a celite column chromatography and eluted with CHCl_3_-MeOH (3:1) to yield 15 fractions. Among these fractions, a distinct and vivid red-purple spot from fr. 6 was isolated, purified and identified as lambetianic acid (LA) with over 98% purity based on spectroscopic analyses such as NMR, MS, and IR as well as the comparison of the data with those reported in the literature [72].

### Cell culture

Human hepatocellular carcinoma(HCC) HepG2, Hep 3B,SK-Hep1 and normal hepatocytes Chang cells were obtained from American Type Culture Collection (ATCC). AMPK α WT and KO mouse embryonic fibroblast (MEF) cells were obtained from Dr. Joohuh Ha. HepG2, SK-Hep1, Chang and MEF cells were cultured in DMEM supplemented with 10% FBS and 1% antibiotic (Welgene, South Korea).

### Cytotoxicity assay

The cytotoxicity of LA was measured by 3-(4,5-dimethylthiazol-2-yl)-2,5-diphenyltetrazolium bromide (MTT) assay. In brief, HepG2, SK-Hep1 and Chang cells (1×10^4^ cells/well) were seeded onto 96-well culture plate and exposed to various concentrations of LA for 24 h. The cells were incubated with MTT (1 mg/mL) (Sigma Chemical) for 2 h and then treated with MTT lysis solution overnight. Optical density (OD) was measured using a microplate reader (Molecular Devices Co., USA) at 570 nm. Cell viability was calculated as a percentage of viable cells in LA treated group versus untreated control.

### Crystal violet assay

For viability and proliferation, crystal violet assay was performed inHepG2 and SK-Hep1 cells. The cells (1×10^5^ cells/well) were seeded onto 6-well culture plate and exposed to various concentrations of LA for 24 h, 48h and 72h. The cells were fixed (4% paraformaldehyde) and stained with crystal violet solution (40% ethanol, 60% PBS and 0.5% crystal violet). Fifteen min later, 1 ml of 10% acetic acid was added to each well, and the absorbance was read at 590 nm using a microplate reader (Molecular Devices Co., USA).

### Cell cycle analysis

HepG2 and SK-Hep1 cells (2 × 10^5^ cells/ml) were treated with LA (0, 10, 20 or 40 μM) for 24 h, washed twice with cold PBS and fixed in 75% ethanol at −20 °C for 24 h. The cells were incubated with RNase A (10 mg/ml) for 1 h at 37°C and stained with PI (50 μg/ml) for 30 min at room temperature in dark. The stained cells were analyzed for the DNA content by FACSCalibur (Becton Dickinson, Franklin Lakes, NJ, USA) using CellQuest Software.

### Annexin V/propidium iodide apoptosis assay

Cell apoptosis assay was performed using the double-staining method of the Annexin-V/PI apoptosis detection kit (BD Pharmingen, Franklin Lakes, NJ, USA) according to the manufacturer's instructions. HepG2 cells (2 × 10^5^ cells/ml) were treated with LA (0, 20 or 40 μM) for 24 h and 48 h. Cells were stained with Annexin V-FITC/PI dye and analyzed immediately by FACSCalibur (Becton Dickinson, Franklin Lakes, NJ, USA). Apoptotic cells were identified as either Annexin V+/PI− staining (early apoptosis) or Annexin V+/PI+ staining (late apoptosis or necrosis cells).

### Measurement of ROS generation

2,7-Dichlorofluorescein diacetate (DCFH-DA) was used to measure the levels of ROS production. HepG2 and SK-Hep1 cells were treated with LA (40 μM) for 3 h, 6 h and then 5 μM DCFH-DA for 30 min at 37°C. Fluorescence intensity was measured by FACSCalibur (Becton Dickinson, Franklin Lakes, NJ, USA).

### Western blotting and co-immunoprecipitation (co-IP)

HepG2 and SK-Hep1 cells (1 × 10^6^ cells/ml) were treated with various concentrations of LA (0, 10, 20 or 40 μM) for 24 h, lyzed in lysis buffer (50 mM Tris–HCl, pH 7.4, 150 mM NaCl, 1% Triton X-100, 0.1% SDS, 1 mM EDTA, 1 mM Na_3_VO_4_, 1 mM NaF, and 1× protease inhibitor cocktail) on ice, and spun down at 14,000×*g* for 20 min at 4°C. The supernatants were collected and quantified for protein concentration by using RC DC protein assay kit (Bio-Rad, Hercules, CA, USA), The protein samples were separated on 4–12% NuPAGE Bis–Tris gels (Novex, Carlsbad, CA, USA) and transferred to a Hybond ECL transfer membrane for detection with antibodies for PARP, cleaved-PARP, Caspase-3, cleaved-Caspase-3, Bcl-2, Bcl-X_L_, phospho-AMPK, AMPK, phospho-ACC, phospho-LKB1, LKB1, PI3K, phospho-AKT, phospho-mTOR, COX-2 (Cell signaling Technology, Beverly, MA, USA) and β-actin (Sigma, St. Louis, MO, USA). For immunoprecipitation experiment, cell lysates were precleared with protein A/G-agarose beads and subsequently incubated for an 1 - 2 h with protein G/A beads covalently coupled with anti-AMPK antibody. Immune complexes were washed four times with cell extraction buffer. Eluted samples or whole cellular lysates were resolved by SDS-PAGE and proteins were detected by Western blotting using the indicated antibodies. Then densitometric analysis was performed using ImageJ software. Unless otherwise specified, actin protein was immunoblotted in order to normalize the quantity of sample protein.

### Statistical analyses

Statistical analysis was performed by Graphpad Prism 5.0 software (GraphPad Software, San Diego, CA, USA). The statistical significance was determined by using one-way ANOVA and Tukey's test or Student's *t*-test. All data were expressed as means ± standard deviation (SD). Statistically significant difference (P<0.05).

## Abbreviations

HCC: hepatocellular carcinoma
LA: lambertianic acid
AMPK: AMP-activated protein kinase
ACC: acetyl-CoA carboxylase
LKB1: liver kinase B1
COX-2: cyclooxygenase-2
mTOR: mammalian target of rapamycin
ROS: reactive oxygen species
NAC: N-acetyl-L-cysteine
MEF: mouse embryonic fibroblasts
MTT: 3-(4,5-dimethylthiazol-2yl)-2,5-diphenyltetrazolium bromide
OD: optical density
FBS: fetal bovine serum
SDS: sodium dodecylsulfate
PBS: phosphate buffered saline
SD: standard deviation
Caspase: cysteine aspartyl-specific protease
PARP: poly (ADP-ribose) polymerase
DAPI: 4’-6-diamidino-2-phenylindole
ECL: enhanced chemiluminescence
AKT: protein kinase B
FACS: fluorescence-activated cell sorting
Bcl-2: B-cell lymphoma 2
DNA: deoxyribonucleic acid
APAF-1: apoptosis-activating factor-1
MAPK: mitogen-activated protein kinase
PPI: protein-protein interaction
